# CytR Homolog of *Pectobacterium carotovorum* subsp. *carotovorum* Controls Air-Liquid Biofilm Formation by Regulating Multiple Genes Involved in Cellulose Production, c-di-GMP Signaling, Motility, and Type III Secretion System in Response to Nutritional and Environmental Signals

**DOI:** 10.3389/fmicb.2017.00972

**Published:** 2017-05-31

**Authors:** M. M. Haque, M. M. H. Oliver, Kamrun Nahar, Mohammad Z. Alam, Hisae Hirata, Shinji Tsuyumu

**Affiliations:** ^1^Department of Environmental Science, Faculty of Agriculture, Bangabandhu Sheikh Mujibur Rahman Agricultural UniversityGazipur, Bangladesh; ^2^Department of Agricultural Engineering, Faculty of Agriculture, Bangabandhu Sheikh Mujibur Rahman Agricultural UniversityGazipur, Bangladesh; ^3^Plant Breeding Division, Bangladesh Agricultural Research InstituteGazipur, Bangladesh; ^4^Faculty of Agriculture, Shizuoka UniversityShizuoka, Japan

**Keywords:** CytR, air-liquid biofilm formation, attachment to plants, cellulose production, c-di-GMP signaling, T3SS, motility, *Pectobacterium carotovorum* subsp. *carotovorum* PC1

## Abstract

*Pectobacterium carotovorum* subsp. *carotovorum* [Pcc (formerly *Erwinia carotovora* subsp. *carotovora*)] PC1 causes soft-rot disease in a wide variety of plant species by secreting multiple pathogenicity-related traits. In this study, regulatory mechanism of *a*ir-*l*iquid (AL) biofilm formation was studied using a *cytR* homolog gene deletion mutant (Δ*cytR*) of Pcc PC1. Compared to the wild type (Pcc PC1), the Δ*cytR* mutant produced fragile and significantly (*P* < 0.001) lower amounts of AL biofilm on *s*alt-*o*ptimized *b*roth plus 2% *g*lycerol (SOBG), yeast peptone dextrose adenine, and also on King’s B at 27°C after 72 h incubation in static condition. The wild type also produced significantly higher quantities of AL biofilm on SOBGMg^–^ (magnesium deprived) containing Cupper (Cu^2+^), Zinc (Zn^2+^), Manganese (Mn^2+^), Magnesium (Mg^2+^), and Calcium (Ca^2+^) compared to the Δ*cytR* mutant. Moreover, the wild type was produced higher amounts of biofilms compared to the mutant while responding to pH and osmotic stresses. The Δ*fliC* (encoding flagellin), *flhD*::Tn5 (encoding a master regulator) and Δ*motA* (a membrane protein essential for flagellar rotation) mutants produced a lighter and more fragile AL biofilm on SOBG compared to their wild counterpart. All these mutants resulted in having weak bonds with the cellulose specific dye (Calcofluor) producing lower quantities of cellulose compared to the wild type. Gene expression analysis using mRNA collected from the AL biofilms showed that Δ*cytR* mutant significantly (*P* < 0.001) reduced the expressions of multiple genes responsible for cellulose production (*bcsA, bcsE*, and *adrA*), motility (*flhD, fliA, fliC*, and *motA*) and type III secretion system (*hrpX, hrpL, hrpA*, and *hrpN*) compared to the wild type. The CytR homolog was therefore, argued to be able to regulate the AL biofilm formation by controlling cellulose production, motility and T3SS in Pcc PC1. In addition, all the mutants exhibited poorer attachment to radish sprouts and AL biofilm cells of the wild type was resistant than stationary-phase and planktonic cells to acidity and oxidative stress compared to the same cells of the Δ*cytR* mutant. The results of this study therefore suggest that CytR homolog is a major determinant of Pcc PC1’s virulence, attachment and its survival mechanism.

## Introduction

*Pectobacterium carotovorum* subsp. *carotovorum* [Pcc (formerly *Erwinia carotovora* subsp. *carotovora*)] is a Gram-negative, necrotrophic and opportunistic phytopathogenic enterobacterium. It is responsible for causing soft-rot on a variety of plant species during cultivation and, in many cases, during post-harvest processing and storage of crops, resulting in significant economic loss (Davidsson et al., 2013). Production of plant cell-wall-degrading enzymes, including pectate lyases (Pel), cellulases (Cel), and proteases (Prt) is the most destructive feature of this pathogen (Toth et al., 2003). Its pathogenicity is also known to be influenced by quorum sensing (Charkowski et al., 2012), motility (Matsumoto et al., 2003; Hossain et al., 2005), type III secretion system (T3SS) encoded by the *h*ypersensitive *r*esponse and *p*athogenicity (*hrp*) gene cluster (Rantakari et al., 2007), gluconate metabolism (Mole et al., 2010), the magnesium/nickel/cobalt transport system (Kersey et al., 2012), biosynthesis of pyrimidine, purine, leucine, or serine (Lee et al., 2013) and biofilm formation (Lee et al., 2013). These pathogenicity factors in Pcc have been shown to be controlled by different regulatory proteins, such as KdgR (Liu et al., 1999), PehR-PehS (Flego et al., 2000), ExpI-ExpR (Lee et al., 2013), GacA-GacS (Cui et al., 2001), RsmA-RsmB-RsmC (Chatterjee et al., 2002a), PmrA-PmrB (Hyytiäinen et al., 2003), and CytR (Matsumoto et al., 2003) in a complex manner.

Biofilms are surface-associated microbial communities, and are able to grow in different abiotic and biotic surfaces (Dolan and Costerton, 2002; McDougald et al., 2012). Biofilm development in bacteria can be divided into three distinct stages (Kaplan, 2010) viz., adhesion, multiplication, and dispersion. In the first stage, planktonic bacteria usually adheres to a biotic or abiotic surface either by physical process including van der Waals forces and electrostatic interactions, or by bacterial surface appendages such as flagella and pili (Rosan and Lamont, 2000). In the second stage, bacteria multiply and communicate with each other through cell–cell communication (auto-inducer) signals (McDougald et al., 2012). At this stage, they also secrete a slimy gelatinous material to form a sizable biomass (Kaplan, 2010). This bacterial biomass comprises of exopolysaccharides [EPSs (mainly cellulose)], proteins, and extracellular DNA (Sutherland, 2001; White et al., 2003; Yap et al., 2005; Liang et al., 2010). The contents ultimately determine the architecture and the stability of a biofilm biomass (Koechler et al., 2015) although their quantities vary depending on the bacterial strains and environmental conditions (Zogaj et al., 2001; Solano et al., 2002; Haque et al., 2012). The sessile bacterial biomass are known to have a hetero-dimensional structure (Oliver et al., 2014) with micro-channels and extended limbs to trap nutrients. The whole assembly acts as a protective barrier against toxins, antimicrobial agents, and predators. In the final stage, a mature biofilm often experiences detachment of some of its own biomass. It is signaled either by the bacteria themselves or caused by external forces (Kaplan, 2010). Such dispersal are considered to be a survival mechanism (O’Toole et al., 2000; Solano et al., 2002; Teitzel and Parsek, 2003; Scher et al., 2005; Haque et al., 2012) as the detached biofilms travel to colonize other sites leading to virulence (Elkins et al., 1999; Yildiz and Schoolnik, 1999; van Houdt and Michiels, 2005; Jahn et al., 2008; Yang et al., 2008; Haque et al., 2012).

Cultured bacteria generally form three types of biofilms in the laboratory: *s*olid-*a*ir-*l*iquid (SAL) interface biofilm, *s*olid-*a*ir (SA) interface biofilm, and *a*ir-*l*iquid (AL) interface biofilm popularly known as pellicle (Zogaj et al., 2001; Solano et al., 2002; Yap et al., 2005; Haque et al., 2009). They have separate genetic, chemical and cultural distinctions (Friedman and Kolter, 2004; Yap et al., 2005; Yang et al., 2008; Haque et al., 2012). Formation of AL biofilm is prevalent in numerous Gram-negative aerobic or facultative aerobic bacteria (Armitano et al., 2014). Flagella, different types of pili, curli fimbriae, type I secretion system, T3SS, cellulose, lipopolysaccharide, and quorum sensing are important for AL biofilm formation (Davey and O’Toole, 2000; Yap et al., 2005; Anriany et al., 2006; Jahn et al., 2008; Liang et al., 2010; Haque et al., 2012; Yamamoto et al., 2012). Nutritional (such as media composition, carbon sources and divalent cations particularly, magnesium, calcium, and iron) and environmental conditions (such as temperature, oxygen tension, chemotaxis, pH, and osmolarity) are also important determinants of the development of AL biofilm in bacteria (Yap et al., 2005; Liang et al., 2010; Yamamoto et al., 2011; Haque et al., 2012; Armitano et al., 2013). Furthermore, numerous transcriptional factors such as AgfD in *Salmonella typhimurium* (Römling et al., 2000), SpoOA in *Bacillus subtilis* (Branda et al., 2001); SlyA, PhoP-PhoQ *t*wo *c*omponent regulatory *s*ystem (TCS) in *Dickeya dadantii* (formerly *Erwinia chrysanthemi*) 3937 (Haque et al., 2009, 2012), Bcam1359 in *Burkholderia cenocepacia* H111 (Fazli et al., 2011), QseC in *Escherichia coli* (Hadjifrangiskou et al., 2012) and RscS in *Vibrio fischeri* (Yip et al., 2006) were also found to have regulated the AL biofilm formation in response to different environmental and nutritional cues.

CytR (*Cyt*idine *R*epressor) is known as a transcriptional repressor of nucleoside uptake and catabolism genes in some bacterial species such as *E. coli* (Valentin-Hansen et al., 1996), *Salmonella enterica* serovar Typhimurium (Thomsen et al., 1999) and *Vibrio cholerae* (Haugo and Watnick, 2002). Biofilm formation in *V. cholerae* is controlled by the CytR through the repression of *vps* genes, which encode enzymes essential for EPS production (Haugo and Watnick, 2002). Watve et al. (2015), on the other hand, reported that CytR is a global positive regulator of competence, type VI secretion and chitinases in *V. cholerae*. In case of Pcc PC1 (formerly EC1) however, the CytR homolog has only been partially characterized by Matsumoto et al. (2003). They showed that the Δ*cytR* mutant is able to reduce the polygalacturonase (Peh) production and increase the production of Pel, Cel, and Prt with respect to its wild counterpart. Swimming motility and the expression of *fliA* (encoding σ^28^) and *fliC* (encoding flagellin) were found to have been dramatically reduced unlike *flhD* (encoding a master regulator) in the Δ*cytR* mutant. Consequently, the virulence was radically reduced in the Δ*cytR* mutant compared to that of the parental strain (Matsumoto et al., 2003). Such discovery was supplemented by Hossain and Tsuyumu (2006) who reported an instance of Pcc PC1 forming SAL biofilm in microtiter plates [made of polyvinyl chloride (PVC)] containing yeast extract peptone broth plus salts of M63 minimal medium at 27°C in static condition. They also showed that SAL biofilm is controlled by motility itself. Despite their efforts, the role of CytR homolog in the formation of AL biofilm in glass test tubes is yet to be quantified under different environmental (i.e., temperature, pH, osmolarity, oxygen tension) and nutritional (i.e., media composition, carbon sources, divalent cations) conditions for Pcc PC1. In addition, the expression of certain genes in this mutant has not been explored with respect to cellulose production.

Cellulose constitutes a gulf of the exopolymeric matrix of AL biofilm in bacteria (Yap et al., 2005; Yang et al., 2008; Haque et al., 2012). It is synthesized by *b*acterial *c*ellulose *s*ynthesis proteins encoded by the *bcs* operons, such as *bcsABCD* and *bcsEFG* (Römling and Galperin, 2015). BcsA is an integral inner membrane protein attached to BcsB, a periplasmic protein. The BcsA contains, among others, a C-terminal fragment that consists of a cyclic-dimeric (3′→5′)-guanosine monophosphate (c-di-GMP) binding PilZ domain (Amikam and Galperin, 2006). The c-di-GMP is known to control numerous cellular functions in bacteria, including biofilm formation, motility and virulence (Yi et al., 2010; Römling et al., 2013). BcsC and BcsD are also required for maximal cellulose production (Saxena et al., 1994). BcsE, BcsF, and BcsG are encoded in the type II *bcs* operons (Römling and Galperin, 2015) and are essential for optimum cellulose synthesis (Solano et al., 2002). The GIL (*G*GDEF *I*-site *l*ike) domain of BcsE is able to bind with c-di-GMP result in additional cellulose production (Fang et al., 2014). The Pcc PC1 genome contains all these *bcsABCD* and *bcsEFG* operons^1^. Nonetheless, we are yet to understand if the CytR homolog of Pcc PC1 is also able to regulate the AL biofilm formation by transcriptional control of the *bcs* genes. The present research aims to explore this area of possibility.

Numerous Gram-negative phytopathogenic bacteria use the T3SS to deliver virulence factors and effectors, such as harpins, avirulence (*avr*) gene and *d*isease-*s*pecific gene *p*roducts (*dsp*) from pathogens into host plant cells (Staskawicz et al., 2001). The T3SS is encoded by *hrp* (*h*ypersensitive *r*esponse and *p*athogenicity) and *hrc* (*h*ypersensitive *r*esponse *c*onserved) genes. Yap et al. (2005) showed that T3SS regulatory (HrpX, HrpY, HrpS, and HrpL) and effector (HrpA and HrpN) proteins are required for AL biofilm formation in *D. dadantii* 3937. A more comprehensive study by Yi et al. (2010) showed that T3SS and biofilm formation on plastic are mediated by phosphodiesterases (PDEs) containing GGDEF and EAL-domain proteins that affect c-di-GMP turnover in *D. dadantii* 3937. The Pcc PC1 genome is known to be containing several GGDEF and EAL-domain proteins^1^. Therefore, the assumption is that such proteins might regulate the biofilm formation in Pcc PC1. Previous studies in this regard, have shed some light on the regulatory role of these genes in case of SAL biofilm only (Yi et al., 2010). Nonetheless, the scientific communities are yet to find out if these genes are able to regulate the AL biofilm formation, or if the CytR homolog of Pcc PC1 can also be affected. This study will contribute toward understanding the role of CytR homolog in cellulose production, while quantifying the expression of *bcs* and T3SS genes in Pcc PC1.

## Materials and Methods

### Bacterial Strains and Growth Media

*Pectobacterium carotovorum* subsp. *carotovorum* [formerly *E. carotovora* subsp. *carotovora* (Pcc])] PC1 [formerly EC1 (wild type)], its derivative strains, Δ*cytR* (aflagellated and non-motile mutant), Δ*fliC* (non-motile and aflagellated mutant), *flhD*::Tn5 (aflagellated and non-motile mutant) and Δ*motA* (flagellated and non-motile mutant) and *D. dadantii* (formerly *Erwinia chrysanthemi*) 3937 (wild type) used in this study has been described earlier by Matsumoto et al. (2003), Haque and Tsuyumu (2005), and Hossain et al. (2005). The strains were freshly grown in yeast extract peptone (YP) medium (1% peptone, 0.5% yeast extract, pH 6.8) at 27°C. Nalidixic acid (30 μg/mL) and kanamycin (50 μg/mL) were added to the media when required, and the optical density (OD) of the culture was measured by a spectrophotometer (Intertech, Inc. Tokyo, Japan) at 660 nm.

### Media, Carbon Source, and Temperature on AL Biofilm Formation

Yeast extract peptone, Luria-Bertani (LB) medium (1% of tryptone, 0.5% of yeast extract, 0.5% of NaCl, pH 7.0), *S*alt-*o*ptimized *b*roth (SOB) plus 2% of *g*lycerol (SOBG) medium (per liter: 20 g of tryptone, 5 g of yeast extract, 0.5 g of NaCl, 0.186 g of KCl, 2.4 g of MgSO_4._7H_2_O and 2% of glycerol), King’s B (KB) medium (per liter: 10 g of peptone, 1.5 g of K_2_HPO_4_, l5 g of MgSO_4._7H_2_O and 15 mL of glycerol), yeast peptone dextrose adenine (YPDA) medium (per liter: 20 g of yeast extract, 40 g of peptone, 40 g of glucose monohydrate, and 80 mg of adenine hemisulfate) and M63 glycerol minimal medium [per liter, 2.5 g of NaCl, 3 g of KH_2_PO_4_, 7 g of K_2_HPO_4_, 2 g of (NH_4_)_2_SO_4_, 0.5 mg of FeSO_4_, 2 g of thiamine hydrochloride, and 2 g of glycerol] were used. In order to quantify the impacts of media and temperature on AL biofilm formation, a single colony of the Pcc PC1 and the Δ*cytR* mutant was grown in shake (180 rpm) culture in YP broth at 27°C until early stationary phase (OD_660_ at 1.0). Afterward, 50 μl of each culture [ca. 10^7^ colony forming unit (CFU)/mL] were suspended in glass test tubes containing 5 mL of the broth. Two separate suspensions were made and incubated at two different temperatures (27 and 37°C, respectively) for 72 h in static condition. In order to study the role of carbon source on AL biofilm formation, 2% glycerol in SOBG was replaced by 2% of glucose, sucrose, or mannitol. Among the media, the Pcc PC1 produced a thick and robust AL biofilm on SOBG broth at 27°C. The SOBG media and 27°C temperature was therefore taken to study the AL biofilm unless otherwise noted in this manuscript.

### Quantification of the Biomass of the AL- and SAL Biofilm

Bacterial strains formed fragile to rigid AL biofilm on SOBG broth. In order to quantify the rigid biomass, samples were prepared as follows: 72 h-old AL biofilms were gently transferred to the fresh test tubes containing 2 mL of distilled water and were vortexed with sterile glass beads. The amount of the biomass in the rigid AL biofilms (OD_600_) were quantified using an UV spectrophotometer (Ultrospec 3000, Pharmacia Biotech, Cambridge, England). In case of fragile AL biofilm, 1 mL of planktonic culture was carefully collected by the pipette and OD_600_ was measured. Afterward, each fragile AL biofilm was mixed with 2 mL of planktonic culture and was vortexed. The OD_600_ of planktonic culture was then subtracted from the OD_600_ of the planktonic culture plus biomass of the fragile AL biofilm. This would provide the amount of fragile biomass present in the AL biofilm.

The SAL biofilms formed at 37°C were also quantified as described in Haque et al. (2012). In brief, the suspension was carefully removed after 72 h and washed the glass test tubes with distilled water. Afterward, 0.05% (w/v) crystal violet was added and incubated for 30 min followed by rinsing with distilled water. Bound crystal violet was eluted using 95% ethanol and the SAL biofilm was quantified (OD_570_) using UV spectrophotometer (Ultrospec 3000, Pharmacia Biotech, Cambridge, England).

### Congo Red and Calcofluor Binding Assays

Congo red and Calcofluor binding assays were carried out as described in Haque et al. (2009, 2012) with a few modifications. Initially, each bacterial strain (Pcc PC1, Δ*cytR*, Δ*fliC, flhD*::Tn5, Δ*motA*, and *D. dadatii* 3937) [used as positive control for the expression of *r*ed, *d*ry *a*nd *r*ough (rdar) phenotype and expression of cellulose] was grown in YP broth overnight at 27°C under shaking condition (180 rpm). Each bacterial culture was serial diluted 1:100 [ca. 10^5^ CFU/mL] and 2 μL of culture were spotted (four spot in each plate) onto SOBG agar plates containing 40 μg/mL of Congo red (Sigma-Aldrich, St. Louis, MO, United States) or 200 μg/mL of Calcofluor white (Sigma-Aldrich, St. Louis, MO, United States). The plates were then incubated at 27°C in static condition, and photographs were taken after 48 h (for Congo red binding). On the other hand, after 48 h incubation, the plates were placed under UV light (366 nm) and photographs were taken for Calcofluor binding.

### Quantification of Cellulose

Amount of cellulose produced in the matrix of the AL biofilms of the bacterial strains (Pcc PC1 Δ*cytR*, Δ*fliC, flhD*::Tn5, and Δ*motA*) were quantified as described in Anriany et al. (2006) with a few modifications. In brief, strains were grown at 27°C in SOBG broth for 3 days in static condition. Then 3 g (wet weight) of AL biofilm masses were gently collected with sterile spatula and were dried by freeze dryer (Eyela, Freeze dryer, FDU-830, Japan). The dry masses were mixed with 4.5 mL of acetic-nitric reagent (8:2:1 acetic acid: nitric acid: distilled water) and boiled for 20 min. The boiled mixture was then centrifuged and the supernatant was discarded. The pellet was transferred to a Corex centrifuged bottles, washed twice with sterile distilled water and dried in a clean bench. The dried pellet was mixed with 150 μL of concentrated H_2_SO_4_ with gentle shaking for 1 h at 27°C. The amount of cellulose was determined by adding 750 μL anthrone (Sigma-Aldrich, St. Louis, MO, United States) reagent (0.2 g in 100 mL H_2_SO_4_). The Avicel cellulose (Sigma-Aldrich, St. Louis, MO, United States) was used as standard this case, and the cellulose were quantified at 620 nm.

### Isolation of RNA

Wild type (Pcc PC1) and Δ*cytR* mutant were grown in SOBG broth until AL biofilm was formed. Total RNA was extracted thereafter from the associated bacteria using an RNA isolation kit (Qiagen, Hilden, Germany) and was subjected to DNase I treatment with the TURBO DNase kit (Ambion, Inc., United States) as instructed by the manufacturer. The purity and concentration of RNA was estimated using a Nanodrop ND-100 spectrophotometer (NanoDrop Technologies, Wilmington, DE, United States).

### Quantitative Reverse Transcription-PCR

Primers (**Table 1**) were designed based on Pcc PC1 DNA sequences that can be retrieved from the following address^1^. cDNA synthesis, verification of the efficiencies of the primers and PCR were carried out as described in Haque et al. (2015). The relative values of transcriptional level were calculated using the ΔΔ*C_T_* method. The abundance of specific gene was initially normalized using 16S rRNA which was shown to be invariant using best keeper (Matsumoto et al., 2003). The relative expression ratio was calculated as the differences between the cycle threshold (*C_T_*) values and was determined using the following equation:

**Table 1:** Primer pairs used in this study.

Primer pairs	Sequence (5′→3′)	Product length (bp)
*bcsA_*forward	TGTAGGCGTGCAACAGGAGAA	—
*bcsA*_reverse	TCGCGGTCGAAACAGTAACGG	315
*bcsE*_forward	ACGGAAGAGCAGCCCATCCTCA	—
*bcsE*_reverse	CGCATGGTGGTCAGCAAGACCT	443
*adrA*_forward	TATAACACCGCGCTGAAGCTG	—
*adrA*_reverse	CCGCCCAATCACATCCGTTT	285
PC1_RS16795_forward	TATGAAGCCCTCGCCAGGTT	—
PC1_RS16795_reverse	TTTCCTGCGCCGAAGTCATC	371
*fliC*_forward	AGTGCTGTCGATAGTGATGG	—
*fliC*_reverse	CGCCAACGATGGTATCTCTC	295
*fliA*_forward	CGAATCCGCGGTTCGATGCT	—
*fliA*_reverse	TCACGCTCTGGCAGGCTTTC	359
*flhD*_forward	TCAATGGCACCGTAACAGCA	—
*flhD*_reverse	ATGGTGTCTTGCAGCGCATT	361
*motA*_forward	GGCCTTACTCTTTCGTGTGATG	—
*motA*_reverse	GATACCAAATGCCGGAAGACC	295
*hrpX*_forward	CTGCCCCAGCTCATCGTGAA	—
*hrpX*_reverse	GCGCGGAAGGTGAAGTGATG	269
*hrpL*_forward	ATCTCATCACCCATTTCCTG	—
*hrpL*_reverse	TACCCTGAAACACATCGAAC	294
*hrpS*_forward	CTTCCTGACTAAAGCGTTGA	—
*hrpS*_reverse	GATGAGATCGACAGTATGCC	271
*hrpA*_forward	AGAACTGGATAGCTTTTGCC	—
*hrpA*_reverse	GGACTTTCTCAGGTTGCATC	196
*hrpN*-forward	GATATTCCGGCTTGCCGAAGA	—
*hrpN*-reverse	CTGTTGTTTAGCGCACTGGAAG	418
*rpoN*_forward	CTGCTGGTACAGCTTTCCCAA	—
*rpoN*_reverse	GGGAATGCTGTCGGTGTTCAG	291
*rsmA*_forward	CCTTGCTAAAAGGCGCACACAG	—
*rsmA*_reverse	TGATCGGCGATGAGGTAACGG	253
*rsmB*_forward	CTGTGGCGGTGATTGACGAA	—
*rsmB*_reverse	CCGAAGGCTCAACCGTATCC	306
16S rRNA_forward	AACTGGCAAGCTAGAGTCTTG	—
16S rRNA_reverse	GCATCGAATTAAACCACATGCT	327

Fold change = 2^-ΔΔC_T_^, where ΔΔ*C_T_* for gene *j* = (*C_T_*_,_*_J_* – *C_T_*_,16SrRNA_)_mutant_ – (*C_T_*_,_*_J_* – *C_T_*_,16SrRNA_)_wildtype._

### Radish Sprouts Attachment Assays

Attachment assays were performed as described in Jahn et al. (2008) with a few modifications. In brief, radish (*Raphanus sativus*) seed (10 g) was collected from Horticulture Research Centre of Bangladesh Agricultural Research Institute, Gazipur, Bangladesh. Seeds were surface sterilized with 75 mL of 1.5% sodium hypochloride for 30 min on a rotary shaker at 200 rpm and was followed by several washes using sterile distilled water. Single colonies of bacterial strain were inoculated into SOBG broth and were grown overnight at 27°C with agitation. It was then centrifuged before each strain was resuspended in sterile distilled water. Approximately 10^4^ CFU/mL bacteria were used. For attachment assays, seeds were germinated in sterile water for 4 days with regular water changes. The sprouts were transferred in sterile 50 mL conical tubes (10 sprouts/tube) and were incubated in bacterial suspension for 4 h at 27°C with gentle shaking (50 rpm). Sprouts were then rinsed three times with sterile distilled water and homogenized. After serial dilution, the homogenate was plated and incubated at 27°C for 24 h for colonies to be enumerated. The experiment was repeated at least three times with at least seven sprouts analyzed per strain each time.

### Stress Tolerance Tests

Stationary (OD_660_ at 1.5) phase cells (grown in shaking condition) and cells-associated with AL biofilm of the wild type and the Δ*cytR* mutant were prepared as described in Haque et al. (2012). In order to prepare the planktonic cells (i.e., cells under the AL biofilm), 1 mL of planktonic culture was carefully collected after 72 h incubation followed by centrifugation. The pellet was then re-suspended in sterile distilled water. In order to test the sensitivities, the stationary-phase, the planktonic cells and the AL biofilm cells (ca. 10^8^ CFU/mL) of the wild type and the Δ*cytR* mutant were separately exposed to acidic pH 4.0 (40 mM citric acid and 20 mM dibasic sodium phosphate, pH 4.0) and oxidative stress (H_2_O_2_ at 10 mM) which corresponds to a lethal concentration (Haque et al., 2009). The suspensions were incubated for a further 2 h at 27°C. The number of CFU was determined by serial dilution and plating onto SOBG agar just prior to inoculation and during every 30 min over a 2-h incubation. The survival of treated cells was normalized to the number of CFU at the beginning of the test.

### Statistical Analysis

All the experiments were conducted in complete randomized design with three replications and repeated at least three times unless otherwise stated. Data were analyzed using Student’s *t*-test of the SAS software system 8.02 (SAS Institute, Cary, NC, United States).

## Results

### Reduced AL Biofilm Formation in the Δ*cytR* Mutant in Different Growth Media

**FIGURE 1:**
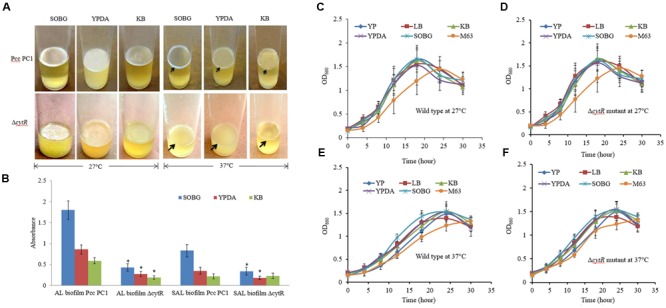
Biofilm formation in different growth media. **(A)** AL- and SAL biofilm formation by the wild type (Pcc PC1) and Δ*cytR* mutant at 27°C and at 37°C, respectively. **(B)** Biomass of AL- and SAL biofilms, measured at 600 and 570 nm, respectively. **(C–F)** Growth rate of the wild type (Pcc PC1) and the Δ*cytR* mutant in different media in shaking condition at 27 and 37°C, respectively. The values are mean and error bars indicate standard deviations ( ± ) of three independent experiments. Asterisks indicate *P* < 0.001 (Student’s *t*-test).

In order to quantify the impacts of media composition and temperature on AL biofilm formation, the Pcc PC1 and the Δ*cytR* mutant cells (ca. 10^7^ CFU/mL) were suspended in five different media (SOBG or KB or LB or YP or M63 glycerol minimal media) and was incubated at two different temperatures (27 or 37°C) in static condition. Selection of five different media and two different temperatures was important to generate information that previous researches did not shed light on. A study by Hossain and Tsuyumu (2006) showed that Pcc PC1 produces SAL biofilm on YP broth plus salts of M63 minimal medium in the wells of microtiter plates made of PVC at 27°C in static condition. They also examined two other media such as YP and LB broth but neither of them produced any SAL or AL biofilms in Pcc PC1. The present study particularly deals with AL biofilm formation in glass test tubes under different environmental conditions which were not examined in any other contemporary researchers dealing with Pcc PC1 and its Δ*cytR* mutant. This study aimed to contribute toward this area of concern, as it was carried out under different media and temperature for a comprehensive result. The results of this study showed that Pcc PC1 formed AL biofilm on SOBG, KB, and YPDA broth after 72 h incubation only at 27°C (**Figure 1A**). In fact, the AL biofilm in SOBG, KB, and YPDA broth did not continue to grow after 7 days (data not shown). However, the AL biofilm in SOBG broth showed signs of detachment at day 7 (data not shown). The AL biofilms formed by Pcc PC1 on the SOBG broth was quite rigid and was denser compared to those on the KB and YPDA broth. On the other hand, biofilms on YPDA and KB by Pcc PC1 were fragile and hence, were dispersed when the samples were disturbed. AL biofilms also acquired a sizable biomass when the glycerol in SOBG was replaced by glucose, sucrose, and mannitol (data not shown). The Δ*cytR* mutant, on the other hand, produced thinner, lighter and more fragile AL biofilm on SOBG, YPDA and KB broth (**Figure 1A**). Quantitative analysis of the biomass at OD_600_ showed that the wild type produced significantly (*P* < 0.001) higher biofilm biomass in SOBG (4.18-fold), YPDA (3.18-fold), and KB (3.10-fold) broth compared to the Δ*cytR* mutant at 27°C (**Figure 1B**). At 37°C however, the Δ*cytR* mutant and its wild counterpart formed only SAL biofilms on SOBG, YPDA, and KB broth (**Figure 1A**). The Pcc PC1 produced a prominent SAL biofilm on SOBG broth compared to those on the YPDA and KB broth (**Figure 1A**). The other three media (YP, LB, and M63 glycerol minimal) did not produce any SAL or AL biofilm for both of these strains under any of the temperatures (27 or 37°C) (data not shown). Similar to the AL biofilms, the amount of SAL biofilm (OD_570_) was also significantly (*P* < 0.001) higher in the wild type on SOBG (2.4-fold) and YPDA (1.9-fold) compared to the Δ*cytR* mutant (**Figure 1B**). Initially however, both wild type (Pcc PC1) and Δ*cytR* mutant cells grew faster in all the broths except in M63 glycerol minimal medium where the growth was slightly delayed both at 27°C (**Figures 1C,D**) and at 37°C (**Figures 1E,F**). In general, the cell growth was slightly delayed in Δ*cytR* mutant compared to that of the wild type in YP broth at both temperatures (**Figures 1C–F**). These results suggested that biofilm formation controlled by the CytR homolog of Pcc PC1 is regulated by media composition and temperature. Bacterial growth might play an important role in this case, although it is very unlikely to be amongst the major determinants.

### Divalent Cations Induced AL Biofilm Formation Controlled by the CytR Homolog

**FIGURE 2:**
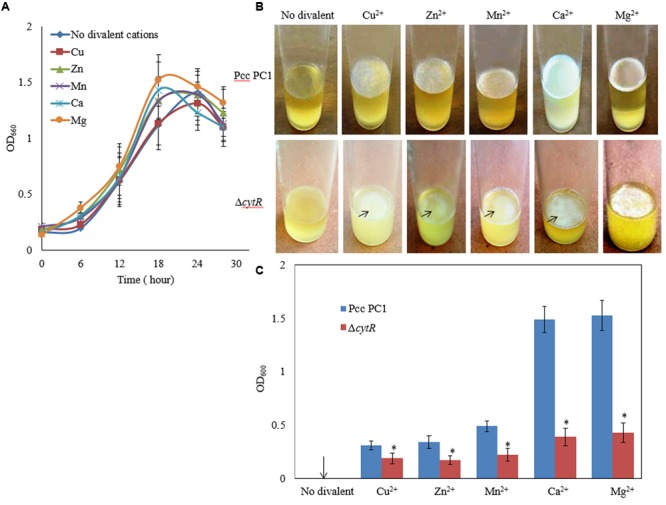
Effect of divalent cations on growth and AL biofilm formation at 27°C. **(A)** Growth of wild type (Pcc PC1) in SOBGMg^–^ (magnesium deprived) and SOBGMg^–^ containing indicated divalent cations. **(B)** AL biofilm formation by wild type and the Δ*cytR* mutant. **(C)** Biomass biofilms. The values are mean and error bars indicate standard deviations ( ± ) of three independent experiments. Asterisks indicate *P* < 0.001 (Student’s *t*-test).

Divalent cations such as Mg^2+^, Ca^2+^, Cu^2+^, Mn^2+^, and Zn^2+^ have been shown to be the inducers and stabilizers of the biofilm formation processes (Song and Leff, 2006; Liang et al., 2010; Haque et al., 2012). However, information regarding the role of such cations in the formation of AL biofilms by Pcc PC1 and its Δ*cytR* mutant was not available in the literature. In this study, the Pcc PC1 (wild type) and Δ*cytR* mutant cells were inoculated in SOBGMg^–^ (magnesium deprived) and SOBGMg^–^ with 0.009 M of Mg^2+^, Ca^2+^, Mn^2+^, Cu^2+^ and Zn^2+^, were incubated at 27°C under shaking (180 rpm) condition. The growth rate was not significantly different between the wild type (**Figure 2A**) and the Δ*cytR* mutant (data not shown) in different incubation period for any divalent cations. Both the wild type and the mutant cells grew quickly and attained its maximum at 18 h of incubation period both in SOBGMg^–^ and SOBGMg^–^ containing 0.009 M Mg^2+^ and Ca^2+^ (**Figure 2A**). In SOBGMg^–^ containing 0.009 M Mn^2+^, Cu^2+^, and Zn^2+^ however, the growth was slow and hence, took 24 h to reach the growth maximum (**Figure 2A**). Interestingly, it was only after 72 h incubation (in static condition) both the strains developed thinner to denser AL biofilm only on SOBGMg^–^ containing the divalent cations (**Figure 2B**). Unlike this, the SOBGMg^–^ broth at 27°C did not show any AL biofilms either in the wild type or in the Δ*cytR* mutant (**Figure 2B**). Compared to the Δ*cytR* mutant, wild type built a denser and robust AL biofilm on SOBGMg^–^ containing Mg^2+^ and Ca^2+^ and a thinner and more fragile AL biofilm on SOBGMg^–^ containing Cu^2+^, Mn^2+^, and Zn^2+^ (**Figure 2B**). When quantified, the production of biomass in the Δ*cytR* mutant was found to be significantly (*P* < 0.001) lower than that of its wild counterpart (**Figure 2C**). These results clearly indicate that the CytR homolog may positively regulate AL biofilm formation in Pcc PC1 responding to divalent cations.

### Reduced AL Biofilm Formation in the Δ*cytR* Mutant in Response to pH

**FIGURE 3:**
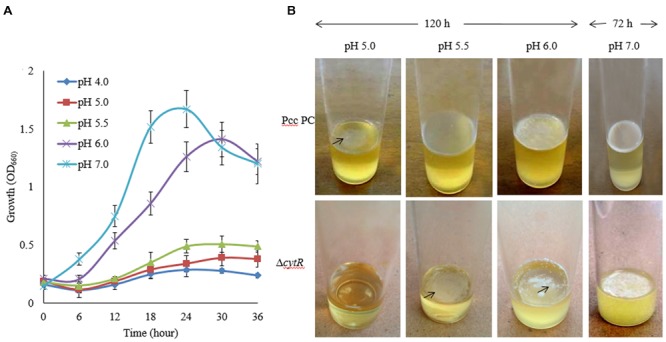
Effect of pH on growth and AL biofilm formation in SOBG broth at 27°C. **(A)** Growth of wild type (Pcc PC1) at indicated pHs. **(B)** AL biofilm formation by the wild type (Pcc PC1) and the Δ*cytR* mutant. The values are mean and error bars indicate standard deviations ( ± ) of three independent experiments.

During early stages of plant infection, pathogenic bacteria often confronts an acidic pH that ranges from pH 4.5 to 6.5 (Grignon and Sentenac, 1991). In order to assess whether Pcc PC1 and Δ*cytR* mutant thrives under acidic conditions, these strains were exposed to acidic pH ranges (**Figure 3A**) using malic acid on SOBG broth at 27°C. The reason behind using malic acid is to stimulate the acidity in plant apoplast that contains malate (Grignon and Sentenac, 1991). The results showed that none of the strains grew at pH 4.5 while showing slower growth at pH 5.0 and pH 5.5 under shaking condition at 27°C. Bacterial cell growth did not differ significantly between the wild type (**Figure 3A**) and the mutant (data not shown) at different incubation period. The wild type formed a delicate AL biofilm on SOBG broth at pH 5.0 and pH 5.5 after 120 h in static condition (**Figure 3B**). On the other hand, Δ*cytR* mutant cells did not grow at the same condition (**Figure 3B**). After 72 h incubation at 27°C, the wild type formed a thinner AL biofilm on SOBG broth at pH 6.0 compared to pH 7.0 (**Figure 3B**). In case of Δ*cytR* mutant, only a few cells aggregated on the top and did not cover the whole surface at pH 6.0 (**Figure 3B**). Our results indicated that acidic pH delayed the AL biofilm formation. The compounds and/or the genes responsible for AL biofilm formation may be not produced or expressed in acidic conditions leading to AL biofilm formation of Pcc PC1.

### Reduced AL Biofilm Formation in the Δ*cytR* Mutant in Response to Osmolarity

**FIGURE 4:**
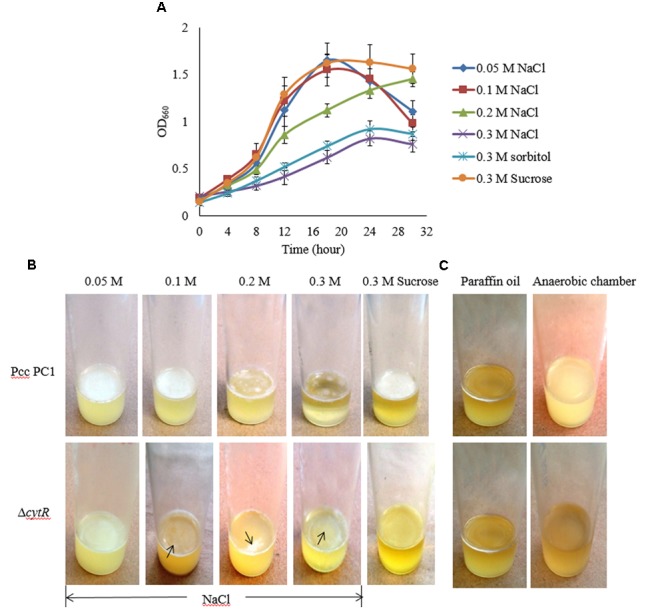
Impacts of osmotic stress on growth and AL biofilm formation in SOBG broth at 27°C. **(A)** Growth of wild type in SOBG containing indicated concentrations of NaCl. **(B)** AL biofilm formation by the wild type (Pcc PC1) and the Δ*cytR* mutant in different concentrations of NaCl. **(C)** No biofilm formed by the wild type and the Δ*cytR* mutant in anaerobic condition. These experiments were repeated at least three times. The values are mean and error bars indicate standard deviations ( ± ) of three independent experiments.

Osmolarity plays a great role in biofilm formation (Solomon et al., 2005; Rinaudi et al., 2006). In order to assess how osmolarity affects the formation of AL biofilm, NaCl and D-sorbitol were used as osmotic agents in the SOBG broth that contains 0.5 g/L NaCl. We however, tested the growth of bacterial cells under different concentrations (0.05, 0.1, 0.2, 0.3 M) of NaCl in shaking condition at 27°C. The results suggest that the growth (OD_660_) of both strains was unaffected due to the changes in osmolarity except for 0.3 M NaCl. Compared to the Δ*cytR* mutant, a firm and intense AL biofilm was developed by the wild type on the SOBG broth containing 0.05 and 0.1 M NaCl after 72 h incubation. Unlike this, 0.3 M NaCl produced small quantities of cells by the wild type and the mutant in shaking condition (**Figure 4A**) and consequently, a few cells were aggregated on the surface of the standing culture (**Figure 4B**). Similar results were also observed when 0.3 M NaCl was replaced by 0.3 M D-sorbitol (data not shown). Conversely, when 0.3 M NaCl/D-sorbitol was replaced by 0.3 M sucrose, both wild type and the Δ*cytR* mutant cells grew rapidly (**Figure 4A**). At this condition, wild type produced a stronger and thicker AL biofilm compared to the Δ*cytR* mutant after 72 h incubation in static condition (**Figure 4B**). These observations suggest that osmotic stress negatively affect AL biofilm formation due to poor growth of the bacterial cells which CytR homolog of Pcc PC1 is able to regulate. This could possibly include the regulation of expression of other components required for AL biofilm formation.

### Oxygen Limits the Formation of AL Biofilm in Pcc PC1 and Δ*cytR* Mutant

Higher oxygen concentrations play an important role in AL biofilm formation (Gerstel and Römling, 2001; Liang et al., 2010). However, this general statement needs to be tested and quantified for the Pcc PC1 and its Δ*cytR* mutant. In order to accomplish this, the wild type and the Δ*cytR* mutant cells (ca. 10^7^ CFU/mL) were suspended in glass test tubes containing 5 mL SOBG broth. It was then sealed with 1.5 mL of sterile liquid paraffin oil and incubated at 27°C for 7 days in static condition. None of the strains formed an AL biofilm although the turbidity of the cells in the broth was increased (**Figure 4C**). Similar results were also observed when wild type and the Δ*cytR* mutant cells (ca. 10^7^ CFU/mL) was suspended in glass test tubes containing 5 mL SOBG broth and incubated at 27°C in anaerobic chamber (Thermo, Inc., Portsmouth, NH, United States) for 7 days (**Figure 4C**). This could be due to the lack of expression of compounds responsible for AL biofilm formation as stated by Gerstel and Römling (2001).

### Motility Itself, But Not Presence of Flagella, Is Required for AL Biofilm Formation

**FIGURE 5:**
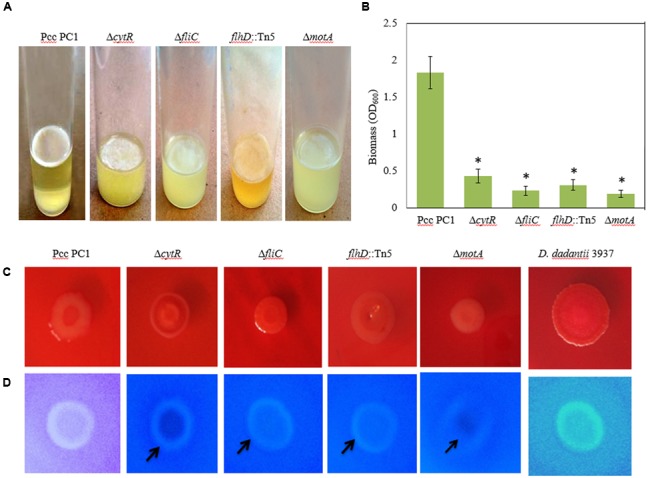
AL Biofilm formation by various strains **(A)**. **(B)** Quantification of the biomass biofilms in the wild type and the mutants. **(C)** Congo red binding (SOBG agar plates containing 40 μg/mL Congo red) abilities of the wild type and the mutants after 48 h incubation. **(D)** Calcofluor binding (SOBG agar plates containing 200 μg/mL Calcofluor) capacities of the wild type and the various mutants after 48 h incubation. These experiments were repeated at least three times. The values are mean and error bars indicate standard deviations ( ± ) of three independent experiments. Asterisks indicate *P* < 0.001 (Student’s *t*-test).

Flagella-mediated motility was shown to play an important role in AL biofilm formation (Jahn et al., 2008; Yang et al., 2008; Haque et al., 2012). On the other hand, Hossain and Tsuyumu (2006) showed that motility, but not the presence of flagella, is required for SAL biofilm formation in microtiter plates of Pcc PC1. Because CytR homolog positively regulates flagella-mediated motility (Matsumoto et al., 2003), we examined whether CytR homolog of Pcc PC1 also regulates AL biofilm by controlling the motility itself. In order to confirm whether the motility itself or the presence of flagella is required for AL biofilm formation on SOBG broth at 27°C in stationary condition, we used three aflagellated and non-motile mutants such as Δ*cytR*, Δ*fliC* and *flhD*::Tn5, and one flagellated and non-motile mutant such as Δ*motA* (essential for flagellar rotation but not required for flagellar assembly) of Pcc PC1. All the mutants produced a lighter and fragile AL biofilm compared to their wild counterpart after 72 h incubation at 27°C (**Figure 5A**). The results also showed that AL biofilm was not increased in the mutants even after longer incubation period (data not shown). When the biofilm biomass was quantified, wild type (Pcc PC1) was found to have produced significantly (*P* < 0.001) more (2.9-, 5.5-, 4.5-, and 9.6-fold) AL biofilms than that of *cytR*, Δ*fliC, flhD*::Tn5, and Δ*motA*, respectively (**Figure 5B**). These data, therefore concludes that motility itself, but not the presence of flagella, is required for AL biofilm formation which the CytR homolog of Pcc PC1 is able to control.

### Reduced Cellulose Production in the Δ*cytR*, Δ*fliC, flhD*::Tn5 and Δ*motA* Mutant

Biofilm producing bacteria develop *r*ed, *d*ry *a*nd *r*ough (rdar) phenotype (also known as rugose/wrinkled phenotype) on Congo red agar plates (Römling, 2005; Haque et al., 2012; Milanov et al., 2015). We hypothesized that CytR homolog, FliC, FlhD, and MotA may affect the expression of rdar phenotype. The results of this study show that the expression of rdar phenotype on Congo red agar plates was indistinguishable between the mutants (such as Δ*cytR*, Δ*fliC, flhD*::Tn5, and Δ*motA*) and the wild type (**Figure 5C**). Therefore, the rdar phenotype may not be controlled by the CytR homolog, FliC, FlhD, and MotA in Pcc PC1.

It is understood that rdar expressing bacteria usually binds with the cellulose specific dye Calcofluor (Zogaj et al., 2001; Solano et al., 2002; Römling, 2005; Uhlich et al., 2006; Steenackers et al., 2012; Milanov et al., 2015). We therefore evaluated whether wild type Pcc PC1 and the mutants (Δ*cytR*, Δ*fliC, flhD*::Tn5, and Δ*motA*) also binds to Calcofluor. The results showed that the wild type (Pcc PC1) had induced bright fluorescence similar to *D. dadantii* 3937, while all the mutants (Δ*cytR*, Δ*fliC, flhD*::Tn5, and Δ*motA*) weakly induced bright fluorescence (**Figure 5D**). This result indicated that wild type Pcc PC1 may produce more cellulose-rich EPS compared to the mutants.

Because cellulose production and AL biofilm formation are correlated in bacteria, we quantified cellulose production in the matrix of the biofilms. The wild type was found to have produced more cellulose (49.7 ± 2.3 ng) than the mutants of Δ*cytR* (21.3 ± 1.7 ng), Δ*fliC* (13.4 ± 2.3 ng), *flhD*::Tn5 (24.3 ± 2.7 ng), and Δ*motA* (9.8 ± 0.8 ng) mutant. According to these results, the increase in Calcofluor binding seemed to have been reflected in the increase of cellulose production. This indicates that the CytR homolog is capable of regulating the AL biofilm formation by controlling both cellulose production and motility in Pcc PC1.

### Reduced Expressions of *bcs* Genes along with *adrA* in the Δ*cytR* Mutant

**FIGURE 6:**
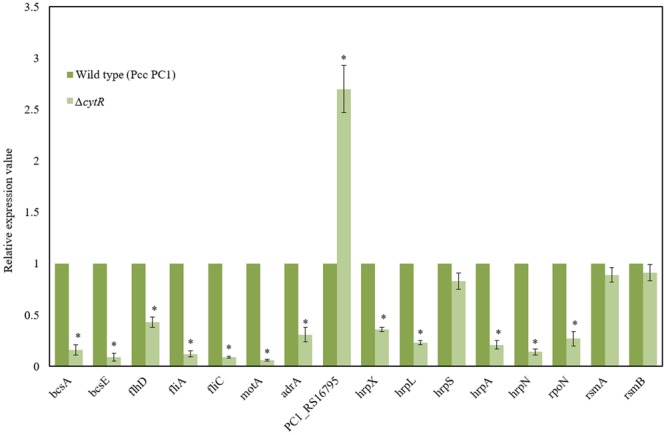
Expressions of various genes in the wild type (Pcc PC1) and Δ*cytR* mutant. Values relative to the mean expression in the wild type were calculated using the ΔΔC_T_ method. Error bars indicate standard deviation ( ± ) for data of three independent experiments. Asterisks indicate *P* < 0.001 (Student’s *t*-test).

*B*acterial *c*ellulose *s*ynthesis (*bcs*) operons, *bcsABZC* and *bcsEFG*, are required for cellulose production leading to AL biofilm formation in *E. coli* and *S. enterica* serovar Typhimurium (Römling et al., 2000; Solano et al., 2002; Da Re and Ghigo, 2006). Homology searches revealed that *bcsA, bcsB, bcsC, bcsE, bcsF*, and *bcsG* of Pcc PC1 had 67.5, 56.5, 50.5, 41.7, 30, and 62.3 similarity to the translated products of *bcsA, bcsB, bcsC, bcsE, bcsF*, and *bcsG* of *E. coli* K-12, respectively, at the amino acid level. We hypothesized that the CytR homolog may affect expression of *bcs* genes in Pcc PC1. In order to test this hypothesis, the expression of *bcsA* (a cellulase synthase) and *bcsE* (maximal cellulose expresser), was measured by quantitative reverse transcription-PCR using mRNA collected from the AL biofilms formed by the wild type and its Δ*cytR* mutant. Expressions of *bcsA* and *bcsE* were significantly (*P* < 0.001) reduced (6.3- and 11.1-fold) in the Δ*cytR* mutant compared to that in the wild type (**Figure 6**). Therefore, the CytR homolog may positively regulates the transcription of *bcs* genes in Pcc PC1.

c-di-GMP is catalyzed by the GGDEF domain proteins present in diguanylate cyclases (DGCs) (Hickman et al., 2005; Ryjenkov et al., 2005) and hydrolyzed by either the EAL or HD-GYP domains present in PDEs (Ryan et al., 2006). The AdrA is a GGDEF domain protein that is able to synthesize the c-di-GMP which binds to the BcsA (Kader et al., 2006; Morgan et al., 2014) and BcsE (Fang et al., 2014). The *adrA* is also required for cellulose biosynthesis in *S. enterica* serovar Typhimurium (Römling et al., 2000; Zogaj et al., 2001). In *D. dadantii* 3937, *ecpB* (a GGDEF-EAL protein) and *ecpC* (a EAL protein) were shown to be negatively regulated the biofilm formation (Yi et al., 2010). The Pcc PC1 genome encodes 17 GGDEF domain proteins, six EAL domain proteins and three GGDEF-EAL domain proteins^1^. In this study, we found that the expression of *adrA* (a GGDEF domain protein) was considerably lower (3.2-fold) in the Δ*cytR* mutant (**Figure 6**) compared to its wild type. However, the expression of PC1_RS16795 (a EAL domain protein) had a dramatic increase (2.7-fold) in the Δ*cytR* mutant compared to the wild type. Thus, CytR homolog may control the GGDEF (AdrA) and EAL (PC1_RS16795) domain proteins in Pcc PC1.

### Reduced Expressions of Motility- and T3SS Genes in the Δ*cytR* Mutant

Because Δ*flhD*, Δ*fliC*, and Δ*motA* produced significantly the lower amount of cellulose than their wild counterpart, in this study, expressions of *flhD, fliA, fliC*, and *motA* were measured by quantitative reverse transcription-PCR using mRNA collected from the AL biofilms formed by the wild type and its Δ*cytR* mutant. Expressions of *flhD* (2.3-fold), *fliC* (11-fold), *fliA* (8.3-fold), and *motA* (16.7-fold) were significantly (*P* < 0.001) decreased in the Δ*cytR* mutant than the wild type (**Figure 6**). These results indicated that CytR homolog positively controls the expressions of *flhD, fliA, fliC*, and *motA* in Pcc PC1.

In *D. dadantii* 3937, T3SS regulatory (*hrpX, hrpY, hrpS*, and *hrpL*), structural (*hrpA, hrcJ*) and effector (*hrpN*) genes are shown to be required for AL biofilm formation (Yap et al., 2005). GGDEF and EAL domain proteins in *D. dadantii* 3937 were also shown to be negatively regulated both the T3SS and biofilm formation (Yi et al., 2010). In our study, we found that GGDEF and EAL domain proteins are also controlled by the CytR homolog of Pcc PC1 (**Figure 6**). Thus, the expression of T3SS genes was examined by quantitative reverse transcription-PCR using mRNA collected from AL biofilms of the wild type and Δ*cytR* mutant. Compared to the wild type, the expression of *hrpX* (2.8-fold), *hrpL* (4.3-fold), *hrpA* (4.8-fold) and *hrpN* (7.1-fold) was found to have significantly (*P* < 0.001) reduced in the Δ*cytR* mutant (**Figure 6**). Thus, CytR homolog is positively controlled the *hrp* genes in Pcc PC1. These results also suggested that CytR homolog is required for *hrpL* expression, which in turn activates the expression of *hrp* genes in the HrpL regulon in Pcc PC1 (Chatterjee et al., 2002b; Yap et al., 2005; Yi et al., 2010).

In our experiment however, the expression of *hrpS* did not differ significantly between the wild type and its Δ*cytR* mutant (**Figure 6**). CytR homolog may affect the expression of *hrpL* through the known *hrpL* regulators, such as RpoN (σ^54^), RsmA (a small RNA-binding protein) or RsmB (a regulatory RNA that binds to and sequesters the negative effect of RsmA on *hrpL* mRNA by forming RsmA–RsmB complex) (Chatterjee et al., 2002b; Yi et al., 2010). The amount of *rsmA* and *rsmB* transcripts in Δ*cytR* mutant was similar to that in the wild type (**Figure 6**). Compared to the wild type, the expression of *rpoN* was significantly (*P* < 0.001) lower (3.7-fold) in the Δ*cytR* mutant (**Figure 6**). Therefore, the CytR homolog may be termed as a regulator of *hrpL* expression which it achieves by altering the expression of *rpoN* in Pcc PC1.

### Reduced Attachments to Radish Sprouts by Δ*cytR*, Δ*fliC, flhD*::Tn5, and Δ*motA* Mutants

**FIGURE 7:**
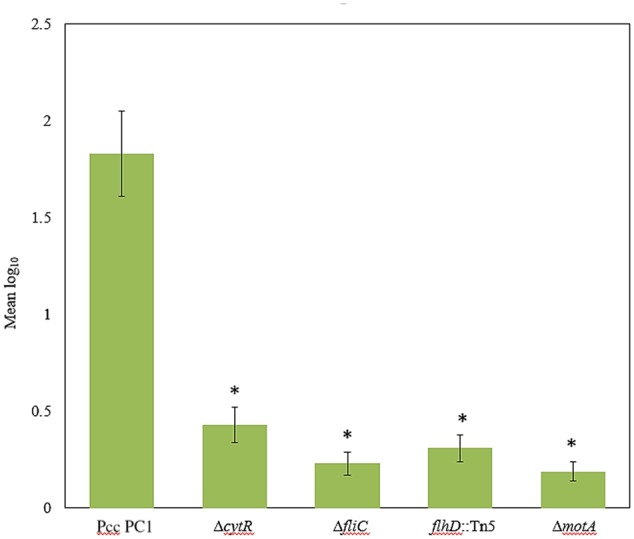
Bacterial attachment to radish sprouts. Radish seeds were germinated in sterile water for 4 days then sprouts were transferred in sterile 50 mL conical tubes (10 sprouts/tube) and incubated in bacterial suspension (ca. 10^4^ CFU/mL) for 4 h at 27°C with gentle shaking at 50 rpm. Sprouts were rinsed three times with sterile distilled water then homogenized. After serial dilution, the homogenate was plated and incubated at 27°C for 24 h then the colonies were enumerated. The experiment was repeated at least three times with at least seven sprouts analyzed per strain each time. The values are mean log_10_ and error bars indicate standard deviation ( ± ) of bacterial cells attached to radish sprouts are presented. Asterisks indicate *P* < 0.001 (Student’s *t*-test).

Because CytR homolog can regulate AL biofilm formation, we hypothesized that similar genes were also required for attachment to plant tissues as well. The Δ*cytR* mutant was significantly reduced (*P* < 0.001) in attachment to radish sprouts compared with the wild type (**Figure 7**). We also tested the Δ*fliC, flhD*::Tn5, and Δ*motA* mutants for the attachment. Compared to the wild type, their attachment was dramatically reduced (*P* < 0.001) in these mutants (**Figure 7**). Thus, CytR homolog, FliC, FlhD, and MotA may regulate both AL biofilm formation in culture and also during attachment to plants in Pcc PC1.

### CytR is Required for Survival in Unfavorable Environments

**FIGURE 8:**
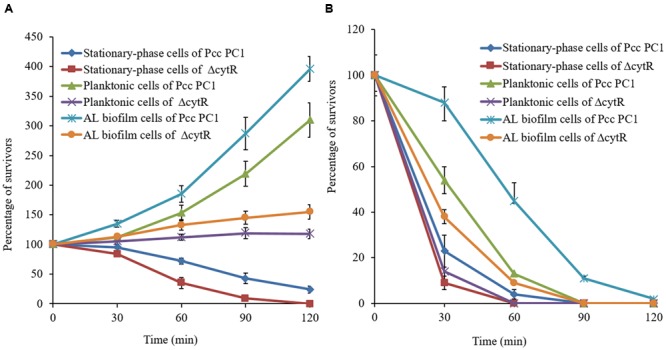
Percentage of cells in bacteria-associated with biofilms, planktonic- and stationary-phase cells surviving a 120-min exposure to acidic (4.0) pH (**A**) and oxidative stress generated by the addition of 10 mM hydrogen peroxide (**B**). Survival rate of the treated cells was normalized to the number of CFU at the beginning of the challenge. Error bars indicate standard deviation ( ± ) for data of three independent experiments.

When Pcc PC1 infects a plant, it confronts an unfavorable environment such as acidity and oxidative stresses. These conditions may play a vital role in survival and expression of the virulence factors (Toth et al., 2003) of any organism. Since biofilms play an important role in the survival of bacteria, we compared the sensitivities of AL biofilms and other cells under conditions of acidity (pH 4.0) and oxidative stress generated by 10 mM H_2_O_2_. Results suggested that the biofilm cells were more resistant than the planktonic and stationary-phase cells (**Figure 8**). The wild type (Pcc PC1) was found to have shown more resistance compared to the Δ*cytR* mutant under acidic pH (**Figure 8A**). All the planktonic- and stationary-phase cells of the Δ*cytR* mutant were killed by 10 mM H_2_O_2_ within the first 60 min, and only 9% of the bacteria-associated with AL biofilm of the Δ*cytR* mutant survived within 60-min exposure time (**Figure 8B**). On the contrary, a 60-min exposure of stationary-phase, planktonic and AL biofilm cells of the wild type (Pcc PC1) against 10 mM H_2_O_2_ resulted in 4, 13, and 45% survival, respectively (**Figure 8B**). Thus, formation of AL biofilm controlled by the CytR homolog may play a great role in the survival of Pcc PC1 under unfavorable environments.

## Discussion

In this article, we demonstrated that CytR homolog of Pcc PC1 is able to positively regulate the AL biofilm formation while responding to different environmental and nutritional signals (**Figures 1**–**5**). The results also showed that the CytR homolog is capable of controlling the expressions of multiple genes required for cellulose biosynthesis, c-di-GMP signaling, motility and T3SS in Pcc PC1 (**Figure 6**). All the mutants were found to diminish their capacity to attachment to radish sprouts (**Figure 7**). We also demonstrated that AL biofilm cells of the wild type was resistant than stationary-phase and planktonic cells to acidity and oxidative stress compared to the same cells of the Δ*cytR* mutant (**Figure 8**). Therefore it can arguably be said that CytR homolog is able to positively control numerous cellular functions in Pcc PC1.

The ability of a bacteria to form biofilms depend on the nutritional conditions (Yap et al., 2005; Hossain and Tsuyumu, 2006; Rinaudi et al., 2006). Divalent cations, particularly Cu^2+^, Ca^2+^, Mn^2+^ and Zn^2+^ and Mg^2+^ play a key role in this process as have been explained for *Shewanella oneidensis* (Liang et al., 2010), *V. cholerae* (Kierek and Watnick, 2003), and *Pseudomonas fluorescens* (Song and Leff, 2006). We also reported that low concentration of Mg^2+^ significantly increase the AL biofilm formation of *D. dadantii* 3937 in a PhoP-PhoQ dependent manner (Haque et al., 2012). The present study also confirms that the formation of AL biofilm controlled by the CytR homolog in Pcc PC1 is activated by the different divalent cations, e.g., Mg^2+^, Ca^2+^, Cu^2+^, Zn^2+^, and Mn^2+^ (**Figure 2B**). Biofilm formation also depends on the environmental conditions such as temperature (Yap et al., 2005), osmotic stress and acidity (Rinaudi et al., 2006). In this study, we observed that high temperature, acidic pH, high osmolarity and anaerobic condition negatively affect the AL biofilm formation controlled by the CytR homolog of Pcc PC1 (**Figures 1, 3**–**5**). Understandably, lower production of cellulose could lead to such scenario, or the genes required for cellulose synthesis were not expressed under those extreme conditions.

Cellulose and curli are two major components of the AL biofilm in *Enterobacteriaece* and their traces can be determined by Congo red binding assays (Steenackers et al., 2012; Milanov et al., 2015). For example, bacterial strains expressing both curli and cellulose leads to the rdar phenotype, only cellulose produces the *p*ink, *d*ry *a*nd *r*ough (pdar) phenotype, and only curli triggers the *b*rown, *d*ry *a*nd *r*ough (bdar) phenotype. A less-prominent phenotype on Congo red agar: ras (*r*ed *a*nd *s*mooth; curli only), pas (*p*ink *a*nd *s*mooth, cellulose only) and bas (*b*rown *a*nd *s*mooth; curli only) has also been reported in some *E. coli* strains (Bokranz et al., 2005). In our study, expression of the rdar phenotype was indistinguishable between the wild type (Pcc PC1) and the mutants (Δ*cytR*, Δ*fliC, flhD*::Tn5, and Δ*motA*) (**Figure 5C**). Compared to the wild type, all the mutants (Δ*cytR*, Δ*fliC, flhD*::Tn5, and Δ*motA*) exhibited weak bond with cellulose specific (Calcofluor) dye (**Figure 5D**). This study also revealed that cellulose production was significantly reduced in the mutants of Δ*cytR*, Δ*fliC, flhD*::Tn5, and Δ*motA* compared to their wild counterpart. The present study also showed that expressions of *flhD, fliA, fliC*, and *motA* are positively controlled by the CytR homolog of Pcc PC1 (**Figure 6**). Thus, fragile and lower AL biofilm formation in these mutants may not be due to only the reduction of motility but also the lower production of cellulose.

Bacterial cellulose synthesis is regulated on both transcriptional and post-transcriptional levels. Expressions of *bcs* genes are controlled by the different regulatory proteins in different bacteria. Transcriptional regulators MlrA and CsgD of *S. enterica* serovar Typhimurium were shown to modulate cellulose biosynthesis indirectly by regulating expression of c-di-GMP synthases DGCs and phosphodiesterases (PGEs) (Römling, 2005; Römling et al., 2013). In *E. coli*, AdrA (a GGDEF domain protein) is responsible for cellulose production (Römling et al., 2000; Zogaj et al., 2001). In our present study showed that expressions of *bcsA, bcsE*, and *adrA* are positively controlled by the CytR homolog of Pcc PC1 (**Figure 6**). Thus, CytR homolog may directly regulate cellulose biosynthesis by transcriptional control of *bcsA, bcsE*, and *adrA* in Pcc PC1.

**FIGURE 9:**
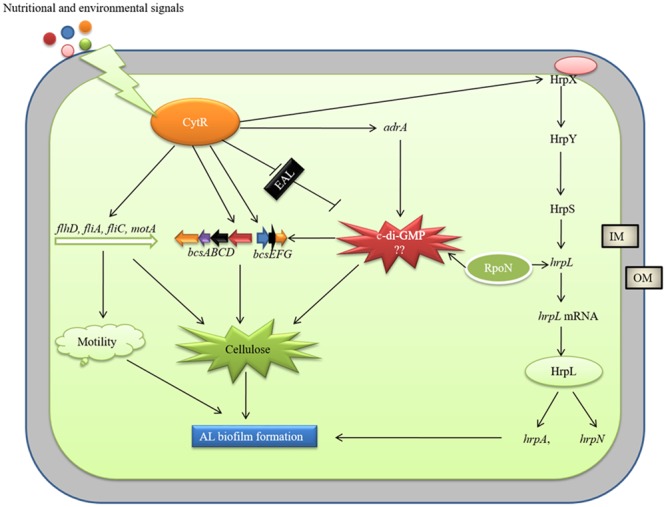
Model of CytR homolog regulatory cascade in Pcc PC1. Environmental (temperature, pH, and osmolarity) and nutritional (growth media and divalent cations) signals modulate CytR expression. The CytR homolog directly controls the expressions of *bcs* operons and *adrA* (a GGDEF domain protein), responsible for cellulose production. The *adrA* may produce c-di-GMP that binds to *bcsA* (Amikam and Galperin, 2006) and *bcsE* (Fang et al., 2014) leading to increase cellulose production. The CytR homolog also regulates the T3SS, essential for AL biofilm formation. The RpoN and HrpL may play a vital role in c-di-GMP regulation of T3SS. However, molecular mechanism by c-di-GMP regulate cellulose production and T3SS yet to be examined. The CytR homolog positively controls the expressions of flagellar formation and rotation genes. These genes are required for cellulose production and motility.

AL biofilm formation is regulated by different regulatory proteins in bacteria. Yang et al. (2008) reported a delay in AL biofilm (pellicle) formation in Δ*gacA* mutant of *D. dadantii* 3937 due to lower expression of *hrpL, dspE* (effector protein), *hrpA*, and *hrpN*. We, in our previous study, reported that PhoP-PhoQ two component system regulates AL biofilm (pellicle) formation by controlling *bcsABCD, adrA* and *fliC* operons but not by controlling the *hrp* genes in *D. dadantii* 3937 (Haque et al., 2012). The present study showed that expressions of *hrpX, hrpL, hrpA*, and *hrpN* are positively controlled by the CytR homolog of Pcc PC1 (**Figure 6**). We also observed that the expression of T3SS genes is regulated by HrpL and possibly by RpoN (**Figure 6**). Based on our results, we therefore, proposed a model in **Figure 9**. It shows that the expression of motility, T3SS and cellulose producing genes are tightly controlled by the CytR homolog of Pcc PC1 through a sophisticated regulatory cascade (**Figure 9**). In our study, the expression of *adrA* (a GGDEF domain protein) is positively controlled by the CytR homolog of Pcc PC1 (**Figure 6**). It was reported that AdrA directly binds to the cellulose synthase complex to deliver c-di-GMP straight to its BcsA (Kader et al., 2006; Morgan et al., 2014). Recently, it was shown that GIL, a new c-di-GMP binding domain protein binds to BcsE which transfers c-di-GMP to BcsA indirectly (Fang et al., 2014). In line with this idea, our results also suggest a strong possibility that c-di-GMP was synthesized by Pcc PC1 and was controlled by the CytR homolog which lead to the production of cellulose and expression of T3SS (**Figure 9**). Similar results have been reported regarding *D. dadantii* 3937 by Yi et al. (2010) who showed that the c-di-GMP turnover was mediated by GGDEF and EAL domain proteins in *D. dadantii* 3937. However, the exact mechanism of c-di-GMP turnover in Pcc PC1 has yet to be deciphered.

AL biofilm formation by plant pathogenic bacteria has been shown to be positively related with their virulence in plants (Jahn et al., 2008; Yang et al., 2008; Haque et al., 2009, 2012). Jahn et al. (2011) reported that symptom development and growth in *Nicotiana benthamiana* are not different between *D. dadantii* 3937 wild type and the *bcsA* (a cellulose synthase) mutant. In this experiment, the degree of maceration (in potato tuber) was significantly increased following inoculation of the wild type compared to the Δ*cytR* mutant (data not shown). However, *in planta* Pel, Cel, and Prt production were indistinguishable between the wild type and the Δ*cytR* mutant (data not shown). Thus, CytR homolog may positively regulate the virulence not by controlling the plant cell-wall-degrading enzymes in Pcc PC1.

Several genes have been shown to be important for both AL biofilm formation and bacterial attachment to plants (Barak et al., 2005; Jahn et al., 2008). We used radish sprouts to test bacterial adherence to plants because Pcc PC1 is known to cause sprout rot of radish. The study showed that the ability of mutants, i.e., Δ*cytR*, Δ*fliC, flhD*::Tn5, and Δ*motA* to adhere themselves to plant tissues was significantly lower than the wild type (**Figure 7**). It has been reported that cellulose synthesized by pathogens and symbionts is essential for host colonization and survival in stress conditions (Augimeri et al., 2015). We have previously reported that bacteria-associated with pellicle/AL biofilm except for aerobically grown logarithmic- and stationary-phase cells of the *D. dadantii* 3937 are more resistant to survival in acidic pH (4.0), oxidative stress and high osmolarity (Haque et al., 2012). In this study, we observed that stationary-phase-, planktonic-, and AL biofilm cells of the wild type are more resistant compared to that in the same cells of the Δ*cytR* mutant under adverse conditions including acidic pH (4.0) and oxidative stress generated by 10 mM H_2_O_2_ (**Figure 8**). These results indicate that cellulose production is linked with the bacterial survival mechanism. Higher cellulose production in the wild type is therefore associated with its increased resistance to acidity and oxidative stress. On the contrary, lower resistance in the Δ*cytR* mutant is linked with lower production of cellulose. This study also reported that planktonic cells are more resistant than stationary-phase cells (**Figure 8**). Similar to Matsumoto et al. (2003), we were unable to complement the restoration of the particular phenotype when *cytR* homolog on pPLAFR_3_ (tetracycline resistant, low-copy-number plasmid) or on pML122 (gentamicin resistant, low-copy-number plasmid) transformed into Δ*cytR* mutant of Pcc PC1. Complementation of the phenotypes in this regulatory gene is yet to be confirmed. We do not know why Δ*cytR* mutant failed to complement the phenotypes. We observed that the CytR homolog of Pcc PC1 regulates numerous cellular functions interacting with different regulatory networks (**Figure 9**). Therefore, plasmid level of expression might be different at native levels and might cause a no complemented phenomena. Yap et al. (2005) reported that the *hrpS* mutant of *D. dadantii* 3937 failed to complement due to a second, unintended spontaneous mutation. It was also reported that subunit interference occurs in homologous proteins leading to failure of complementation (Leonhartsberger et al., 2000).

The AL biofilm formation in Pcc PC1 is an emergent property. We are yet to know what role AL biofilms play in the expression of virulence of Pcc PC1. In this study, we observed that bacteria-associated with AL biofilms are more resistance to acidic pH and oxidative stress. Thus, formation of AL biofilm in Pcc PC1 may protect the cells against unfavorable environment. Moreover, the matrix of AL biofilm of Pcc PC1 is composed of cellulose which is commercially available as a wound dressing material (Abeer et al., 2014). Further research endeavors with Pcc PC1 could look into this area of possibilities.

## Conclusion

The present study has demonstrated that CytR homolog of Pcc PC1 positively regulate AL biofilm formation in culture responding to environmental and nutritional signals. This study has clearly demonstrated that CytR homolog of Pcc PC1 positively regulate bacterial attachment to radish sprouts as it arguably controls the expression of numerous genes involved in cellulose production, c-di-GMP signaling, motility and the T3SS. The outcomes of this study are expected to contribute toward understanding the virulence factors, attachment to plant tissues and survival of Pcc PC1 in unfavorable environments.

## Author Contributions

All authors listed, have made substantial, direct and intellectual contribution to the work. All authors read and approved the final manuscript.

## Conflict of Interest Statement

The authors declare that the research was conducted in the absence of any commercial or financial relationships that could be construed as a potential conflict of interest.

## Footnotes

1 https://www.ncbi.nlm.nih.gov/nuccore/CP001657.1

## References

[B1] Abeer M. M. Mohd Amin M. C. Martin C. (2014). A review of bacterial cellulose-based drug delivery system: their biochemistry, current approaches and future prospects. *J. Pharm. Pharmacol.* 66 1047–1061. 10.1111/jphp.12234 24628270

[B2] Amikam D. Galperin M. Y. (2006). PilZ domain is part of the bacterial c-di-GMP binding protein. *Bioinformatics* 22 3–6. 10.1093/bioinformatics/bti739 16249258

[B3] Anriany Y. Sanu S. N. Wessels K. R. McCann L. M. Joseph S. W. (2006). Alteration of the roguse phenotype in waaG and ddhC mutants of *Salmonella enterica* serovar Typhimurium DT104 is associated with inverse production of curli and cellulose. *Appl. Environ. Microbiol.* 72 5002–5012. 10.1128/AEM.02868-05 16820499 PMC1489332

[B4] Armitano J. Méjean V. Jourlin-Castelli C. (2013). Aerotaxis governs floating biofilm formation in *Shewanella oneidensis*. *Environ. Microbiol.* 15 3108–3118. 10.1111/1462-2920.12158 23751053

[B5] Armitano J. Méjean V. Jourlin-Castelli C. (2014). Gram-negative bacteria can also form pellicles. *Environ. Microbiol. Rep.* 6 534–544. 10.1111/1758-2229.12171 25756106

[B6] Augimeri R. V. Varley A. J. Strap J. L. (2015). Establishing a role for bacterial cellulose in environmental interactions: lessons learned from diverse biofilm-producing *Proteobacteria*. *Front. Microbiol.* 6:1282 10.3389/fmicb.2015.01282 PMC4646962 26635751

[B7] Barak J. D. Gorski L. Naraghi-Arani P. Charkowski A. O. (2005). *Salmonella enterica* virulence genes are required for bacterial attachment to plant tissue. *Appl. Environ. Microbiol.* 71 5685–5691. 10.1128/AEM.71.10.5685-5691.2005 16204476 PMC1265987

[B8] Bokranz W. Wang X. Tschape H. Romling U. (2005). Expression of cellulose and curli fimbriae by *Escherichia coli* isolated from the gastrointestinal tract. *J. Medical Microbiol.* 54 1171–1182. 10.1099/jmm.0.46064-0 16278431

[B9] Branda S. S. González-Pastor J. E. Ben-Yehuda S. Losick R. Kolter R. (2001). Fruiting body formation by *Bacillus subtilis*. *Proc. Natl. Acad. Sci. USA* 98 11621–11626. 10.1073/pnas.191384198 11572999 PMC58779

[B10] Charkowski A. Blanco C. Condemine G. Expert D. Franza T. Hayes C. (2012). The role of secretion systems and small molecules in soft-rot *Enterobacteriaceae* pathogenicity. *Ann. Rev. Phytopathol.* 50 425–450. 10.1146/annurev-phyto-081211-173013 22702350

[B11] Chatterjee A. Cui Y. Chatterjee A. K. (2002a). RsmA and the quorum-sensing signal, N-[3-oxohexanoyl]-L-homoserine lactone, control the levels of rsmB RNA in *Erwinia carotovora* subsp. carotovora by affecting its stability. *J. Bacteriol.* 184 4089–4095. 12107125 10.1128/JB.184.15.4089-4095.2002 PMC135201

[B12] Chatterjee A. Cui Y. Chatterjee A. K. (2002b). Regulation of *Erwinia carotovora* hrpLEcc (sigma-LEcc), which encodes an extracytoplasmic function subfamily of sigma factor required for expression of the HRP regulon. *Mol. Plant Microbe Interact.* 9 971–980. 10.1094/MPMI.2002.15.9.971 12236604

[B13] Cui Y. Chatterjee A. Chatterjee A. K. (2001). Effects of the two-component system comprising GacA and GacS of *Erwinia carotovora* subsp. carotovora on the production of global regulatory rsmB RNA, Extracellular Enzymes, and HarpinEcc. *Mol. Plant Microbe Interact.* 14 516–526. 10.1094/MPMI.2001.14.4.516 11310739

[B14] Da Re S. Ghigo J. M. (2006). A CsgD-independent pathway for cellulose production and biofilm formation in *Escherichia coli*. *J. Bacteriol.* 188 3073–3087. 10.1128/JB.188.8.3073-3087.2006 16585767 PMC1447019

[B15] Davey M. E. O’Toole G. A. (2000). Microbial biofilms: from ecology to molecular genetics. *Microbiol. Mol. Biol. Rev.* 64 847–867. 10.1128/MMBR.64.4.847-867.2000 11104821 PMC99016

[B16] Davidsson P. R. Kariola T. Niemi O. Palva E. T. (2013). Pathogenicity of and plant immunity to soft rot pectobacteria. *Front. Plant Sci.* 4:191 10.3389/fpls.2013.00191 PMC3678301 23781227

[B17] Dolan R. Costerton W. (2002). Biofilms: survival mechanisms clinical microorganisms. *Clin. Microbiol. Rev.* 15 167–193. 10.1128/CMR.15.2.167-193.2002 11932229 PMC118068

[B18] Elkins J. M. Hassett D. J. Stewart P. S. Schweizer H. P. McDermott T. R. (1999). Protective role of catalase in *Pseudomonas aeruginosa* biofilm resistance to hydrogen peroxide. *Appl. Environ. Microbiol.* 65 4594–4600. 10508094 10.1128/aem.65.10.4594-4600.1999 PMC91612

[B19] Fang X. Ahmad I. Blanka A. Schottkowski M. Cimdins A. Galperin M. Y. (2014). GIL, a new c-di-GMP binding protein domain involved in regulation of cellulose synthesis in enterobacteria. *Mol. Microbiol.* 93 439–452. 10.1111/mmi.12672 24942809 PMC4116459

[B20] Fazli M. O’Connell A. Nilsson M. Niehaus K. Dow J. M. Givskov M. (2011). The CRP/FNR family protein Bcam1349 is a c-di-GMP effector that regulates biofilm formation in the respiratory pathogen *Burkholderia cenocepacia*. *Mol. Microbiol.* 82 327–341. 10.1111/j.1365-2958.2011.07814.x 21883527

[B21] Flego D. Marits R. Eriksson A. R. Kõiv V. Karlsson M. B. Heikinheimo R. (2000). A two-component regulatory system, pehR-pehS, controls endopolygalacturonase production and virulence in the plant pathogen *Erwinia carotovora* subsp. carotovora. *Mol. Plant Microbe Interact.* 13 447–455. 10.1094/MPMI.2000.13.4.447 10755308

[B22] Friedman L. Kolter R. (2004). Genes involved in matrix formation in *Pseudomonas aeruginosa* PA14 biofilms. *Mol. Microbiol.* 51 675–690. 10.1046/j.1365-2958.2003.03877.x 14731271

[B23] Gerstel U. Römling U. (2001). Oxygen tension and nutrient starvation are major signals that regulate agfD promoter activity and expression of the multicellular morphotype in *Salmonella* typhimurium. *Environ. Microbiol.* 3 638–648. 10.1046/j.1462-2920.2001.00235.x 11722544

[B24] Grignon C. Sentenac H. (1991). pH and ionic conditions in the apoplast. *Annu. Rev. Plant Physiol.* 42 103–128. 10.1146/annurev.pp.42.060191.000535

[B25] Hadjifrangiskou M. Gu A. P. Pinkner J. S. Kostakioti M. Zhang E. W. Greene S. E. (2012). Transposon mutagenesis identifies uropathogenic *Escherichia coli* biofilm factors. *J. Bacteriol.* 194 6195–6205. 10.1128/JB.01012-12 22984258 PMC3486386

[B26] Haque M. M. Hirata H. Tsuyumu S. (2012). Role of PhoP-PhoQ two-component system in pellicle formation, virulence and survival in harsh environments of *Dickeya dadantii* 3937. *J. Gen. Plant Pathol.* 78 176–189. 10.1007/s10327-012-0372-z

[B27] Haque M. M. Hirata H. Tsuyumu S. (2015). SlyA regulates motA and motB, virulence and stress-related genes under conditions induced by the PhoP-PhoQ system in *Dickeya dadantii* 3937. *Res. Microbiol.* 166 467–475. 10.1016/j.resmic.2015.05.004 26027774

[B28] Haque M. M. Kabir M. S. Aini L. Q. Hirata H. Tsuyumu S. (2009). SlyA, a MarR family transcriptional regulator, is essential for virulence in *Dickeya dadantii* 3937. *J. Bacteriol.* 191 5409–5419. 10.1128/JB.00240-09 19542281 PMC2725626

[B29] Haque M. M. Tsuyumu S. (2005). Virulence, resistance to magainin II and expression of pectate lyase are controlled by the PhoP-PhoQ two-component regulatory system responding to pH and magnesium in *Erwinia chrysanthemi* 3937. *J. Gen. Plant Pathol.* 71 47–53.19 10.1007/s10327-004-0158-z

[B30] Haugo A. J. Watnick P. I. (2002). *Vibrio cholerae* CytR is a repressor of biofilm development. *Mol. Microbiol.* 45 471–483. 10.1046/j.1365-2958.2002.03023.x 12123457 PMC2515492

[B31] Hickman J. W. Tifrea D. F. Harwood C. S. (2005). A chemosensory system that regulates biofilm formation through modulation of cyclic diguanylate levels. *Proc. Natl. Acad. Sci. U.S.A.* 102 14422–14427. 10.1073/pnas.0507170102 16186483 PMC1234902

[B32] Hossain M. M. Shibata S. Aizawa S.-I. Tsuyumu S. (2005). Motility is an important determinant for pathogenesis of *Erwinia carotovora* subsp. *carotovora*. *Physiol. Mol. Plant Pathol.* 66 134–143. 10.1016/j.pmpp.2005.06.001

[B33] Hossain M. M. Tsuyumu S. (2006). Flagella-mediated motility is required for biofilm formation by *Erwinia carotovora* subsp. *carotovora*. *J. Gen. Plant Pathol.* 72 34–39. 10.1007/s10327-005-0246-8

[B34] Hyytiäinen H. Sjöblom S. Palomäki T. Tuikkala A. Palva E. T. (2003). The PmrA-PmrB two-component system responding to acidic pH and iron controls virulence in the plant pathogen *Erwinia carotovora* ssp. *carotovora*. *Mol. Microbiol.* 50 795–807. 10.1046/j.1365-2958.2003.03729.x 14617142

[B35] Jahn C. E. Selimi D. A. Barak J. D. Charkowski A. O. (2011). The *Dickeya dadantii* biofilm matrix consists of cellulose nanofibres, and is an emergent property dependent upon the type III secretion system and the cellulose synthesis operon. *Microbiology* 157 2733–2744. 10.1099/mic.0.051003-0 21719543

[B36] Jahn C. E. Willis D. K. Charkowski A. O. (2008). The flagellar sigma factor FliA is required for *Dickeya dadantii* virulence. *Mol. Plant Microbe Interact.* 21 1431–1442. 10.1094/MPMI-21-11-1431 18842093

[B37] Kader A. Simm R. Gerstel U. Morr M. Römling U. (2006). Hierarchical involvement of various GGDEF domain proteins in rdar morphotype development of *Salmonella enterica* serovar Typhimurium. *Mol. Microbiol.* 60 602–616. 10.1111/j.1365-2958.2006.05123.x 16629664

[B38] Kaplan J. B. (2010). Biofilm dispersal: mechanisms, clinical implications, and potential therapeutic uses. *J. Dent. Res.* 89 205–218. 10.1177/0022034509359403 20139339 PMC3318030

[B39] Kersey C. M. Agyemang P. A. Dumenyo C. K. (2012). CorA, the magnesium/nickel/cobalt transporter, affects virulence and extracellular enzyme production in the soft rot pathogen *Pectobacterium carotovorum*. *Mol. Plant Pathol.* 13 58–71. 10.1111/j.1364-3703.2011.00726.x 21726393 PMC6638878

[B40] Kierek K. Watnick P. I. (2003). The *Vibrio cholerae* O1390-antigen polysaccharide is essential for Ca2+-depedent biofilm development in sea water. *Proc. Natl. Acad. Sci. U.S.A.* 100 14357–14362. 10.1073/pnas.2334614100 14614140 PMC283596

[B41] Koechler S. Farasin J. Cleiss-Arnold J. Arséne-Ploetze F. (2015). Toxic metal resistance in biofilms: diversity of microbial responses and their evolution. *Res. Microbiol.* 10 764–773. 10.1016/j.resmic.2015.03.008 25869223

[B42] Lee D. H. Lim J. A. Lee J. Roh E. Jung K. Choi M. (2013). Characterization of genes required for the pathogenicity of *Pectobacterium carotovorum* subsp. *carotovorum* Pcc21 in Chinese cabbage. *Microbiology* 159 1487–1496. 10.1099/mic.0.067280-0 23676432 PMC3749726

[B43] Leonhartsberger S. Ehrenreich A. Böck A. (2000). Analysis of the domain structure and the DNA binding sites of the transcriptional activator FhlA. *Eur. J. Biochem.* 267 3672–3684. 10.1046/j.1432-1327.2000.01399.x 10848985

[B44] Liang Y. Gao H. Chen J. Dong Y. Wu L. He Z. (2010). Pellicle formation in *Shewanella oneidensis*. *BMC Microbiol.* 10:291 10.1186/1471-2180-10-291 PMC2995470 21080927

[B45] Liu Y. Jiang G. Cui Y. Mukherjee A. Ma W. L. Chatterjee A. (1999). kdgREcc negatively regulates genes for pectinases, cellulase, protease, harpinEcc, and a global RNA regulator in *Erwinia carotovora* subsp. *carotovora*. *J. Bacteriol.* 181 2411–2422. 10198003 10.1128/jb.181.8.2411-2421.1999 PMC93665

[B46] Matsumoto H. Muroi H. Umehara M. Yoshitake Y. Tsuyumu S. (2003). Peh production, flagellum synthesis, and virulence reduced in *Erwinia carotovora* subsp. *carotovora* by mutation in a homologue of cytR. *Mol. Plant Microbe Interact.* 16 389–397. 10.1094/MPMI.2003.16.5.389 12744509

[B47] McDougald D. Rice S. A. Barraud N. Steinberg P. D. Kjelleberg S. (2012). Should we stay or should we go: mechanisms and ecological consequences for biofilm dispersal. *Nat. Rev. Microbiol.* 10 39–50. 10.1038/nrmicro2695 22120588

[B48] Milanov D. S. Prunic B. Z. Velhner M. J. Pajic M. L. Cabarkapa I. S. (2015). Rdar morphotype- a resting stage of some *Enterobacteriaceae*. *Food Feed Res.* 42 43–50. 10.5937/FFR1501043M

[B49] Mole B. Habibi S. Dangl J. L. Grant S. R. (2010). Gluconate metabolism is required for virulence of the soft-rot pathogen *Pectobacterium carotovorum*. *Mol. Plant Microbe Interact.* 23 1335–1344. 10.1094/MPMI-03-10-0067 20636105

[B50] Morgan J. L. McNamara J. T. Zimmer J. (2014). Mechanism of activation of bacterial cellulose synthase by cyclic di-GMP. *Nat. Struct. Mol. Biol.* 21 489–496. 10.1038/nsmb.2803 24704788 PMC4013215

[B51] Oliver M. M. H. Hewa G. A. Pezzaniti D. (2014). Bio-fouling of subsurface type drip emitters applying reclaimed water under medium soil thermal variation. *Agril. Water Manage.* 133 12–23. 10.1016/j.agwat.2013.10.014

[B52] O’Toole G. Kaplan H. B. Kolter R. (2000). Biofilm formation as microbial development. *Annu. Rev. Microbiol.* 54 49–79. 10.1146/annurev.micro.54.1.49 11018124

[B53] Rantakari A. Virtaharju S. Taira S. Palva E. T. Saarilahti H. T. Romantschuk M. (2007). Type III secretion contributes to the pathogenesis of the soft-rot pathogen *Erwinia carotovora*: partial characterization of the hrp gene cluster. *Mol. Plant Microbe Interact.* 14 962–968. 10.1094/MPMI.2001.14.8.962 11497468

[B54] Rinaudi L. Fujishige N. A. Hirsch A. M. Banchio E. Zorreguieta A. Giordano W. (2006). Effects of nutritional and environmental conditions on *Sinorhizobium meliloti* biofilm formation. *Res. Microbiol.* 15 867–875. 10.1016/j.resmic.2006.06.002 16887339

[B55] Römling U. (2005). Characterization of the rdar morphotype, a multicellular behavior in *Enterobacteriaceae*. *Cell Mol. Life Sci.* 62 1234–1246. 10.1007/s00018-005-4557-x 15818467 PMC11139082

[B56] Römling U. Galperin M. Y. (2015). Bacterial cellulose biosynthesis: diversity of operons, subunits, products and functions. *Trends Microbiol.* 23 545–557. 10.1016/j.tim.2015.05.005 26077867 PMC4676712

[B57] Römling U. Galperin M. Y. Gomelsky M. (2013). Cyclic di-GMP: the first 25 years of a universal bacterial second messenger. *Microbiol. Mol. Biol. Rev.* 77 1–52. 10.1128/MMBR.00043-12 23471616 PMC3591986

[B58] Römling U. Rohde M. Olsen A. Normark S. Reinkoster J. (2000). agfD, the checkpoint of multicellular and aggregative behaviour in *Salmonella* typhimurium regulates at least two independent pathways. *Mol. Microbiol.* 36 10–23. 10.1046/j.1365-2958.2000.01822.x 10760159

[B59] Rosan B. Lamont R. J. (2000). Dental plague formation. *Microbes Infect.* 2 1599–1607. 10.1016/S1286-4579(00)01316-2 11113379

[B60] Ryan R. P. Fouhy Y. Lucey J. F. Crossman L. C. Spiro S. He Y. W. (2006). Cell-cell signaling in Xanthomonas campestris involves an HD-GYP domain protein that functions in cyclic di-GMP turnover. *Proc. Natl. Acad. Sci. U.S.A.* 103 6712–6717. 10.1073/pnas.0600345103 16611728 PMC1458946

[B61] Ryjenkov D. A. Tarutina M. Moskvin O. V. Gomelsky M. (2005). Cyclic diguanylate is a ubiquitous signaling molecule in bacteria: insights into biochemistry of the GGDEF protein domain. *J. Bacteriol.* 187 1792–1798. 10.1128/JB.187.5.1792-1798.2005 15716451 PMC1064016

[B62] Saxena I. M. Kudlicka K. Okuda K. Brown R. M. Jr. (1994). Characterization of genes in the cellulose-synthesizing operon (*acs* operon) of *Acetobacter xylinum*: implications for cellulose crystallization. *J. Bacteriol.* 176 5735–5752. 10.1128/jb.176.18.5735-5752.1994 8083166 PMC196778

[B63] Scher K. Römling U. Yaron S. (2005). Effect of heat, acidification, and chlorination on *Salmonella enterica* serovar Typhimurium cells in a biofilm formed at the air-liquid interface. *Appl. Environ. Microbiol.* 71 1163–1168. 10.1128/AEM.71.3.1163-1168.2005 15746314 PMC1065136

[B64] Solano C. García B. Valle J. Berasain C. Ghigo J. M. Gamazo C. (2002). Genetic analysis of *Salmonella* enteritidis biofilm formation: critical role of cellulose. *Mol. Microbiol.* 43 793–808. 10.1046/j.1365-2958.2002.02802.x 11929533

[B65] Solomon E. B. Brendan A. N. Sapers G. M. Annous B. A. (2005). Biofilm formation, cellulose production, and curli biosynthesis by *Salmonella* originating from produce, animal, and clinical sources. *J. Food Protect.* 68 906–912. 10.4315/0362-028X-68.5.906 15895720

[B66] Song B. Leff L. G. (2006). Influence of magnesium ions on biofilm formation by *Pseudomonas fluorescens*. *Microbiol. Res.* 161 355–361. 10.1016/j.micres.2006.01.004 16517137

[B67] Staskawicz B. Mudgett M. B. Dangl J. L. Galan J. E. (2001). Common and contrasting themes of plant and animal diseases. *Science* 292 2285–2289. 10.1126/science.1062013 11423652

[B68] Steenackers H. Hermans K. Vanderleyden J. Keersmaecker D. (2012). An overview on *Salmonella* biofilms: an overview on occurrence, structure, regulation and eradication. *Food Res. Int.* 45 502–531. 10.1016/j.foodres.2011.01.038

[B69] Sutherland I. W. (2001). Biofilm exopolysaccharides: a strong and stick frame-work. *Microbiology* 147 3–9. 10.1099/00221287-147-1-3 11160795

[B70] Teitzel G. M. Parsek M. R. (2003). Heavy metal resistance of biofilm and planktonic *Pseudomonas aeruginosa*. *Appl. Environ. Microbiol.* 69 2313–2320. 10.1128/AEM.69.4.2313-2320.2003 12676715 PMC154819

[B71] Thomsen L. E. Pedersen M. Norregaard-Madsen M. Valentin-Hansen P. Kallipolitis B. H. (1999). Protein-ligand interaction: grafting of the uridine-specific determinants from the CytR regulator of *Salmonella* typhimurium to *Escherichia coli* cytR. *J. Mol. Biol.* 288 165–175. 10.1006/jmbi.1999.2668 10329134

[B72] Toth I. K. Bell M. C. Holeva M. C. Birch P. R. J. (2003). Soft rot erwiniae: from genes to genomes. *Mol. Plant Pathol.* 4 17–30. 10.1046/j.1364-3703.2003.00149.x 20569359

[B73] Uhlich G. A. Cooke P. H. Solomon E. B. (2006). Analyses of the red-dry-rough phenotype of an *Escherichia coli* O157:H7 strain and its role in biofilm formation and resistance to antimicrobial agents. *Appl. Environ. Microbiol.* 72 2564–2572. 10.1128/AEM.72.4.2564-2572.2006 16597958 PMC1449024

[B74] Valentin-Hansen P. Sogaard-Andersen L. Pedersen H. (1996). A flexible partnership: the CytR anti-activator and the cAMP-CRP activator protein, comrades in transcription control. *Mol. Microbiol.* 20 461–466. 10.1046/j.1365-2958.1996.5341056.x 8736525

[B75] van Houdt R. Michiels C. W. (2005). Role of bacterial cell surface structures in *Escherichia coli* biofilm formation. *Res. Microbiol.* 156 626–633. 10.1016/j.resmic.2005.02.005 15950122

[B76] Watve S. S. Thomas J. Hammer B. K. (2015). CytR is a global positive regulator of competence, type VI secretion, and chitinases in *Vibrio cholerae*. *PLoS ONE* 10:e0138834 10.1371/journal.pone.0138834 PMC4581735 26401962

[B77] White A. Gibson D. L. Collinson S. K. Banser P. A. Kay W. W. (2003). Extracellular polysaccharides associated with thin aggregative fimbriae of *Salmonella enterica* serovar Enteritidis. *J. Bacteriol.* 185 5398–5407. 10.1128/JB.185.18.5398-5407.2003 12949092 PMC193744

[B78] Yamamoto K. Arai H. Ishii M. Igarashi Y. (2011). Trade-off between oxygen and iron acquisition in bacterial cells at the air-liquid interface. *FEMS Microbiol. Ecol.* 77 83–94. 10.1111/j.1574-6941.2011.01087.x 21395624

[B79] Yamamoto K. Arai H. Ishii M. Igarashi Y. (2012). Involvement of flagella-driven motility and pili in *Pseudomonas aeruginosa* colonization at the air-liquid interface. *Microbes Environ.* 27 320–323. 10.1264/jsme2.ME11322 22353768 PMC4036044

[B80] Yang S. Peng Q. Zhang Q. Yi X. Choi C. J. Reedy R. M. (2008). Dynamic regulation of GacA in type III secretion, pectinase gene expression, pellicle formation, and pathogenicity of *Dickeya dadantii* (*Erwinia chrysanthemi* 3937). *Mol. Plant Microbe Interact.* 21 133–142. 10.1094/MPMI-21-1-0133 18052890

[B81] Yap M.-N. Yang C.-H. Barak J. D. Jahn C. E. Charkowski A. O. (2005). The *Erwinia chrysanthemi* type III secretion system is required for multicellular behavior. *Mol. Microbiol.* 187 639–648. 10.1128/jb.187.2.639-648.2005 PMC543537 15629935

[B82] Yi X. Yamazaki A. Biddle E. Zeng Q. Yang C.-H. (2010). Genetic analysis of two phosphodiesterases reveals cyclic diguanylate regulation of virulence factors in *Dickeya dadantii*. *Mol. Microbiol.* 77 787–800. 10.1111/j.1365-2958.2010.07246.x 20584146

[B83] Yildiz F. H. Schoolnik G. K. (1999). *Vibrio cholerae* O1 EI Tor: identification of a gene cluster required for the rugose colony type, exopolysaccharide production, chlorine resistance, and biofilm formation. *Proc. Natl. Acad. Sci. U.S.A.* 96 4028–4033. 10.1073/pnas.96.7.4028 10097157 PMC22414

[B84] Yip E. S. Geszvain K. DeLoney-Marino C. R. Visick K. L. (2006). The symbiosis regulator rscS controls the syp gene locus, biofilm formation and symbiotic aggregation by *Vibrio fischeri*. *Mol. Microbiol.* 62 1586–1600. 10.1111/j.1365-2958.2006.05475.x 17087775 PMC1852533

[B85] Zogaj X. Nimtz M. Rohde M. Bokranz W. Römling U. (2001). The multicellular morphotypes of *Salmonella* typhimurium and *Escherichia coli* produce cellulose as the second component of the extracellular matrix. *Mol. Microbiol.* 39 1452–1463. 10.1046/j.1365-2958.2001.02337.x 11260463

